# Data-based reconstruction of complex geospatial networks, nodal positioning and detection of hidden nodes

**DOI:** 10.1098/rsos.150577

**Published:** 2016-01-06

**Authors:** Ri-Qi Su, Wen-Xu Wang, Xiao Wang, Ying-Cheng Lai

**Affiliations:** 1 School of Electrical, Computer, and Energy Engineering, Arizona State University, Tempe, AZ 85287, USA; 2 School of Biological and Health Systems Engineering, Arizona State University, Tempe, AZ 85287, USA; 3 Department of Systems Science, School of Management and Center for Complexity Research, Beijing Normal University, Beijing 100875, People’s Republic of China

**Keywords:** time-series analysis, network reconstruction, geospatial network, compressive sensing

## Abstract

Given a complex geospatial network with nodes distributed in a two-dimensional region of physical space, can the locations of the nodes be determined and their connection patterns be uncovered based solely on data? We consider the realistic situation where time series/signals can be collected from a single location. A key challenge is that the signals collected are necessarily time delayed, due to the varying physical distances from the nodes to the data collection centre. To meet this challenge, we develop a compressive-sensing-based approach enabling reconstruction of the full topology of the underlying geospatial network and more importantly, accurate estimate of the time delays. A standard triangularization algorithm can then be employed to find the physical locations of the nodes in the network. We further demonstrate successful detection of a hidden node (or a hidden source or threat), from which no signal can be obtained, through accurate detection of all its neighbouring nodes. As a geospatial network has the feature that a node tends to connect with geophysically nearby nodes, the localized region that contains the hidden node can be identified.

## 1. Introduction

Complex geospatial networks with components distributed in real geophysical space are an important part of the modern infrastructure. Examples include large-scale sensor networks and various subnetworks embedded in the Internet. For such a network, often the set of active nodes depends on time: the network can be regarded as static only on a relatively short time scale. For example, in response to a certain breaking news event, a communication network within the Internet may emerge, but the network will dissolve itself after the event and its impacts fade away. The connection topologies of such networks are usually unknown but in certain applications, it is desirable to uncover the network topology and to *determine the physical locations of various nodes* in the network. Suppose time series or signals can be collected from the nodes. Due to the distributed physical locations of the nodes, the signals are time delayed. Is it possible to uncover the network topology, estimate the time delays embedded in the signals from different nodes, and then determine their physical locations? Another issue is the existence of hidden nodes that cannot be directly accessed. Can the existence of a hidden node be ascertained and its location be determined?

**Figure 1.**
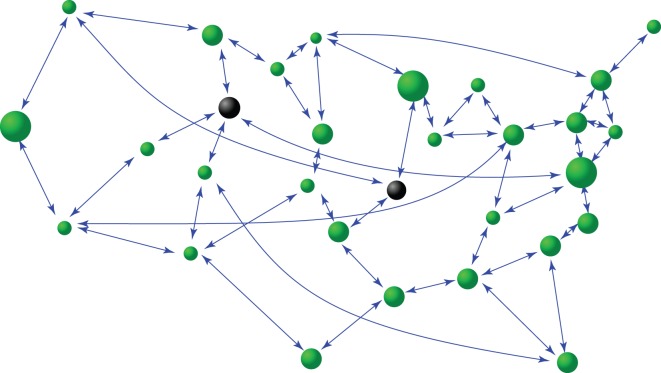
A schematic illustration of a complex geospatial network. The connection topology, the positions of the nodes in the physical space and nodal dynamical equations are unknown *a priori*, but only time series from the nodes can be collected at a single node in the network (e.g. a data-collecting centre). The challenges are to reconstruct the dynamical network, to locate the precise position of each node and to detect hidden nodes, all based solely on time series with inhomogeneous time delays. The green circles denote ‘normal’ nodes and the dark circles indicate hidden nodes.

Figure 1 illustrates a geospatial network. Assume there is a monitoring centre that collects data from nodes at various locations, but their precise geospatial coordinates are unknown. The normal nodes are coloured in green. There are also hidden nodes that can potentially be the sources of threats (e.g. those represented by dark circles). The challenging task is to determine the network topology and to locate the hidden nodes, based on time series or data only.

Data-based reconstruction of complex networks in general is deemed to be an important problem and has attracted continuous interest, where the goal is to uncover the full topology of the network based on simultaneously measured time series [1–26]. For instance, methodology was proposed to estimate the network topology controlled by feedback or delayed feedback [8,17]. Network connectivity can be reconstructed from the collective dynamical trajectories using response dynamics [7,16]. The approach of random phase resetting was introduced to reconstruct the details of the network structure [14]. For neuronal systems, there was a statistical method to track the structural changes [20,22]. While many of these previous works required complete or partial information about the intrinsic dynamics of the nodes and their coupling functions, completely data-driven and model-free methods exist. For example, the global climate network was reconstructed using the mutual information method, enabling global energy and information flow in the network to be studied [25]. The sampling bias of DNA sequences in viruses from different regions can be used to reveal the geospatial topologies of influenza networks [26]. Network structure can also be obtained by calculating the causal influences among the time series based on the Granger causality method [5,23], the transfer entropy method [21] or the method of inner composition alignment [15]. However, such causality-based methods are unable to reveal information about the nodal dynamical equations. In addition, there were regression-based methods [27] for systems identification based on, for example, the least-squares approximation through the Kronecker-product representation [28], which would require large amounts of data.

In this paper, we develop a compressive-sensing-based framework [29–32] as a potential solution to estimating time delay and detecting hidden nodes in complex geospatial networks. To be able to fully reconstruct dynamical systems using only time series data is possible because the dynamics of many natural and man-made systems are determined by smooth enough functions that can be approximated by finite series expansions. The task then becomes that of estimating the coefficients in the series representation of the vector field governing the system dynamics. In general, the series can contain high-order terms, and the total number of coefficients to be estimated can be quite large. This is in general a challenging problem. However, if most coefficients are zero (or negligible), the vector constituting all the coefficients will be sparse. The problem of sparse vector estimation can then be solved by the paradigm of compressive sensing [29,30,32–34] that reconstructs a sparse signal from limited observations. Since the observation requirements can be relaxed considerably as compared to those associated with conventional signal reconstruction schemes, compressive sensing has evolved into a powerful technique to reconstruct sparse signal from small amounts of observations that are much less than those required in conventional approaches.

Compressive sensing has recently been introduced to the field of network reconstruction for discrete time and continuous time nodal dynamics [35,36], for evolutionary game dynamics [37], for detecting hidden nodes [38,39], for predicting and controlling synchronization dynamics [40], and for reconstructing spreading dynamics based on binary data [41]. Differing from these existing works, the focus of the present work is on estimating time delays of the dynamics at various nodes using time series collected from a single location. While there were previous methods of finding time delays in complex dynamical systems, for example, based on synchronization [42], Bayesian estimation [43] and correlation between noisy signals [19], our compressive-sensing-based method provides an alternative approach that has the advantages of generality, high efficiency, low data requirement and applicability to large networks. We demonstrate that our method can yield estimates of the nodal time delays with reasonable accuracy. After the time delays are obtained, the actual geospatial locations of various nodes can be determined by using, for example, a standard triangular localization method [44]. We expect these results to be useful for applications such as locating sensors in wireless networks and identifying/detecting/anticipating potential geospatial threats [45], an area of importance and broad interest.

## 2. Results

Compressive sensing aims to solve the following convex optimization problem:

2.1 $$\min\;∥\text{a}{∥}_{1}\;\text{subject to}\;\text{G}\cdot \text{a}=\text{X},$$

where **a** is a sparse vector to be determined, **G** is a (known) random projection matrix, **X** is a measurement vector that can be constructed from the available data, and $∥\text{a}{∥}_{1}=\sum_{i=1}^{N}|{\text{a}}_{i}|$ is the *L*_1_ norm of vector **a**. Compressive sensing is a paradigm of high-fidelity signal reconstruction using only sparse data [29,30,32–34], which was originally developed to solve the problem of transmitting massive datasets, such as those collected from large-scale sensor arrays. In particular, because of the high dimensionality, direct transmission of such datasets would require broad bandwidth. However, there are common situations where the datasets are sparse. For example, say a dataset of *N* points is represented by an *N*×1 vector **a**, where *N* is a large integer. Since **a** is sparse, most of its entries are zero and only a small number of *k* entries are non-zero, where *k*≪*N*. One can use a Gaussian random matrix **G** of dimension *M*×*N* to obtain an *M*×1 vector **X**: **X**=**G**⋅**a**, where *M*∼*k*. Because the dimension of **X** is much smaller than that of the original vector **a**, transmitting **X** would require much smaller bandwidth, provided that **a** can be reconstructed at the receiver end of the communication channel.

For our problem of reconstructing complex geospatial networks with time delay, the task is to formulate the problem into the standard compressive sensing form of equation (2.1). This can indeed be done, for example, for oscillator networks with weighted coupling and inhomogeneous time delays [46,47]. After obtaining the time delays, a standard triangular localization method [44] can be employed to locate a large portion of nodes in the network, given that the locations of a small subset of nodes are known. A hidden node can also be detected (see Methods).

### 2.1 Reconstruction of geospatial networks based on compressive sensing

To be concrete, we present results for continuous-time oscillator networks with time-delayed couplings [46–48], where for every link, the amount of delay is proportional to the physical distance of this link. The oscillator network models have been widely used in studying neuron activity [47] and ecology [48]. In general, such a system is described by

2.2 $${\dot{\text{x}}}_{i}={\text{F}}_{i}[{\text{x}}_{i}(t)]+\sum_{j=1,j\neq i}^{N}{\text{W}}_{ij}[{\text{x}}_{j}(t-\tau_{ij})-{\text{x}}_{i}(t)],$$

for *i*=1,…,*N*, where ${\text{x}}_{i}\in R^{m}$ is the *m*-dimensional state variable of node *i* and **F**_*i*_[**x**_*i*_(*t*)] is the vector field for its isolated nonlinear nodal dynamics. Consider a link *l*_*ij*_ connecting nodes *i* and *j*, and the interaction weight is given by the *m*×*m* weight matrix ${\text{W}}_{ij}\in R^{m\times m}$ with its element $w_{ij}^{p,q}$ representing the coupling from the *q*th component of node *j* to the *p*th component of node *i*. For simplicity, we assume only one component of $w_{ij}^{p,q}$ is non-zero and denote it as *w*_*ij*_. The associated time delay is denoted as *τ*_*ij*_. For a modern geospatial network, the speed of signal propagation is quite high in a proper medium (e.g. optical fibre). The time delay can thus be assumed to be small and we can use the Taylor expansion to express the delay coupling terms in the networked dynamical system to the first order, e.g. $x_{i}(t-\tau_{ji})\approx x_{i}(t)-\tau_{ji}{\dot{x}}_{i}$, where ${\dot{x}}_{i}$ is the time derivative. When the coupling function between any pair of nodes is linear, in a suitable mathematical basis constructed from time-series data, ${\tilde{\text{g}}}^{\left(\gamma \right)}[{\text{x}}_{i}(t)]$, the coupling and time-delayed terms, together with the nodal dynamical equations, can be expanded into series, and our goal is to estimate all the expansion coefficients. Using our formulation of the compressive sensing framework (see Methods), equation (2.2) can then be written in the following compact form:

2.3 $${\dot{\text{x}}}_{i}(t)=\sum_{\gamma }{\tilde{\alpha }}^{\left(\gamma \right)}\cdot {\tilde{\text{g}}}^{\left(\gamma \right)}[{\text{x}}_{i}(t)]+\sum_{j=1,j\neq i}^{N}\left[{\text{b}}_{ij}{\text{x}}_{j}(t)+{\text{c}}_{ij}{\dot{\text{x}}}_{j}(t)\right],$$

which is a set of linear equations for data collected at different time *t*. The coefficients associated with the nodal dynamical equations, the network link-weights and time delays are contained in the coefficients $\left\{{\tilde{\alpha }}^{\left(\gamma \right)}\right\}$, {**b**_*ij*_} and {**c**_*ij*_}, respectively. The amount of data required depends on the system size and the order of the series expansions, which can be small as compared with the dimension of the coefficient vectors for a properly chosen mathematical base.

After obtaining the time delays, we proceed to determining the actual positions of all nodes. If time series are collected simultaneously from all nodes at the data-collecting node, the estimated coupling delay *τ*_*ij*_ associated with the link *l*_*ij*_ is proportional to the physical distance *d*_*ij*_=*d*_*ji*_ of the link. However, in reality, strictly synchronous data collection is not possible. For example, if the signals are collected at a location *s* outside the network with varying time delays *τ*_*si*_, the estimated delays associated with various links in the network are no longer proportional to the actual distances. As we explain in Methods, the varying delays due to asynchronous data collection can be cancelled and the distances can still be estimated as *d*_*ij*_=(*c*/2)(*τ*_*ij*_+*τ*_*ji*_), where *τ*_*ij*_ is the signal delay associated with node *j* from the reconstruction of node *i*, vice versa for *τ*_*ji*_, and *c* is the signal propagation speed.

When the mutual distances between nodes have been estimated, we can determine the actual locations of the nodes, for example, by using the standard triangular localization algorithm [44]. This method requires that the positions of *N*_B_ reference nodes be known, the so-called beacon nodes. Starting from the beacon nodes, the triangulation algorithm makes use of the distances to these reference nodes to calculate the Cartesian coordinates of the detected nodes. The beacon node set can then be expanded with the newly located nodes. Nodes that are connected to the new beacon set, each with more than three links in two-dimensional space, can be located. The process continues until the locations of all nodes have been determined, or no new nodes can be located (see Methods). The choice of the proper initial beacon set to fully reconstruct the network depends on the network topology. For example, for a scale-free network, we can choose nodes that were firstly added during the process of network generation as the initial beacon set, thereby guaranteeing that all nodes can be located using the procedure. An empirical rule is then to designate the largest degree nodes as the initial beacon node set.

Our numerical experiments are set up as follows. We assume all nodes are distributed in a two-dimensional square, or a three-dimensional cube of unit length. The network topology can be either scale-free [49] or random [50], and the network size can be varied. For proof of principle, we consider coupled nonlinear oscillator networks by placing, at each node, a nonlinear oscillator, e.g. the Rösseler oscillator, mathematically described by the following set of three first-order differential equations: $\left[\dot{x},\dot{y},\dot{z}]=[-y-z,x+0.2y,0.2x+z(x-0.2)\right]$. The coupling weights are asymmetric and uniformly distributed in the interval [0.1, 0.5]. We assign a small threshold to the estimated weight as *w*_0_=0.05 (somewhat arbitrary), where if the estimated weight is larger (smaller) than *w*_0_, the corresponding link is regarded as existent (non-existent). We have *d*_*ij*_=*c*⋅*τ*_*ij*_=*c*⋅*τ*_*ji*_ and choose *c* to be 100 (arbitrarily). To be specific, we choose linear coupling functions and assume that, for a pair of connected nodes, the interaction occurs between the *z*-variable of one node and the *x*-variable of another. The time series used to reconstruct the whole network system are acquired by integrating the coupled delayed differential equation system [51] with step size 5×10^−5^. The vector fields of the nodal dynamics are expanded into a power series of order *l*_*x*_+*l*_*y*_+*l*_*z*_≤3. The derivatives required for the compressive sensing formulation are approximated from time series by the standard first-order Gaussian method. To quantify the data requirement, we define *R*_*m*_ as the ratio of the number of data points used to the total number of unknown coefficients to be estimated. The beacon nodes are chosen to be those having the largest degrees in the network, and their positions are assumed to be known.

**Figure 2.**
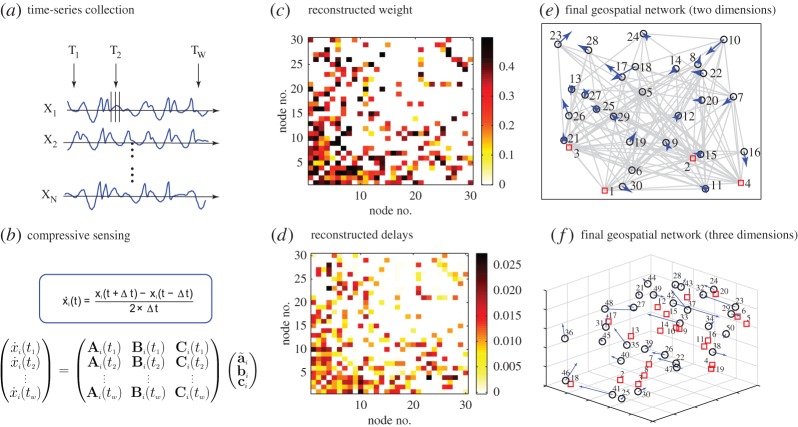
Illustration of our method to reconstruct a complex geospatial network from time series. (*a*) For any node, time series of its dynamical variables are collected at *w* different instants of time. (*b*) The corresponding derivatives are approximated using the standard first-order Gaussian method, which are needed in constructing the compressive sensing equations. (*c*,*d*) An example of link weights and time delays obtained from the reconstructed coefficient vectors, respectively. (*e*) Given the positions of four beacon nodes (marked as red rectangles), the locations of the remaining nodes (marked as black circles) are determined using the standard triangularization method. The blue arrows indicate the estimation errors, which point from the actual to the estimated positions. The various coupling terms are illustrated using grey lines. There are in total 30 nodes in the network, connecting with each other via the scale-free topology. The average outgoing degree is five. The amount of data used is *R*_*m*_=0.5. (*f*) Following the same procedure, a geospatial network of 50 nodes which are distributed in a three-dimensional cube of unit length is reconstructed. There are 20 beacon nodes and the normalized amount of data used is *R*_*m*_=0.55. For clarity, the links between these nodes are not shown.

Figure 2 summarizes the major steps required for reconstructing a complex geospatial network using compressive sensing. For illustrative purpose, we first use a network of *N*=30 nodes that are connected with each other in a scale-free manner and are randomly distributed in a two-dimensional square. Oscillatory time series are collected from each node, from which compressive sensing equations can be obtained, as shown in figure 2*a*,*b*. The reconstructed coefficients for the nodal dynamical equations, as explained in Methods, contain the coupling weights *B*_*ij*_=*w*_*ij*_ and the delay terms *C*_*ij*_=−*w*_*ij*_×*τ*_*ij*_. The links with reconstructed weights larger than the threshold *w*_0_ are regarded as actual (existent) links, for which the time delays *τ*_*ij*_ can be estimated as *τ*_*ij*_=−*C*_*ij*_/*w*_*ij*_. Repeating this procedure for all nodes, we can determine the weighted adjacency matrix (that defines the network topology) and the time delay matrix. The estimated adjacency matrix and the time delays are displayed in figure 2*c*,*d*, respectively, which match well with those of the actual network. We note that the reconstructed time delays are symmetric with respect to the link directions, as shown in figure 2*d*, which is correct as they depend only on the corresponding physical distances. With the estimated time delays, we choose the four largest degree nodes, nodes 1 to 4, as the beacon nodes, so that the locations of all remaining nodes can be determined. The fully reconstructed geospatial network is shown in figure 2*e*, where the red rectangles indicate the locations of the beacon nodes. The black circles denote the actual locations of the remaining nodes and the heads of the blue arrows indicate their estimated positions (shorter arrows mean higher estimation accuracy). The amount of data used is relatively small: *R*_*m*_=0.5.

We further test our method in three dimensions. Using the same conditions for all nodes that are randomly distributed in a three-dimensional cube of unit length, we perform reconstruction on a scale-free network with 50 nodes, as shown in figure 2*f*. We follow the same method to estimate the connection weights and time delays for connected pairs, and then use triangulation localization to locate all nodes in the three-dimensional space. We employ 20 beacon nodes and use *R*_*m*_=0.55, because a higher dimensional system usually requires more reference nodes and larger data amount.

### 2.2 Performance analysis with respect to weight and time delay estimates

The performance of our compressive-sensing-based approach to reconstructing geospatial networks can be assessed by calculating the errors in the estimated weights and time delays. We define two types of errors: those associated with non-zero terms (existing links, denoted as *W*_nz_ and *D*_nz_ for weight and time delay, respectively), and those associated with zero terms (non-existing links, *W*_z_ and *D*_z_). In particular, *W*_nz_ is the error between the estimated and the true weight for an existent link, normalized by the latter, while *W*_z_ is the average absolute error associated with the original zero terms in the coefficients. Similar meanings hold for *D*_nz_ and *D*_z_.

**Figure 3.**
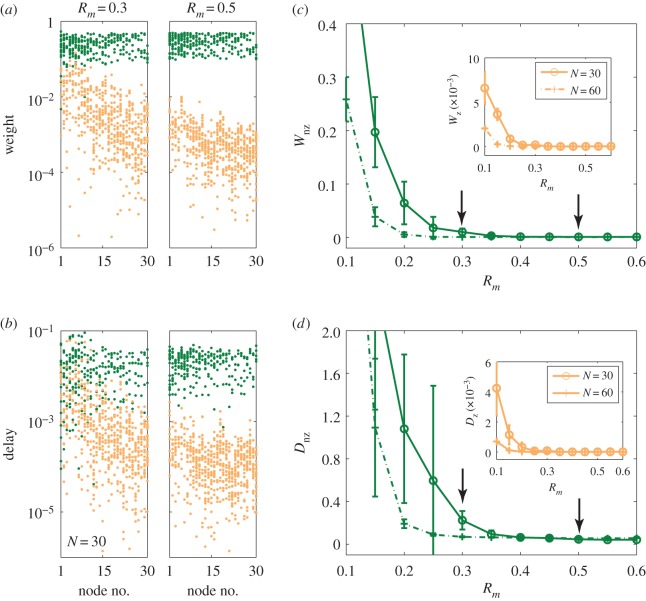
Error analysis of network reconstruction and delay time estimation. (*a*) Predicted incoming coupling strengths for all nodes for *R*_*m*_=0.3 and *R*_*m*_=0.5 on a logarithmic scale. The green and orange dots represent the weights of existent and non-existent links, respectively. (*b*) Predicted coefficients for the non-zero delay coupling terms *C*_*ij*_, marked as green dots, in comparison with the estimated values for zero terms (marked as orange dots). (*c*) Errors in the estimates of the coupling weights associated with existent and non-existent links, *W*_nz_ and *W*_z_ (inset), respectively, versus the data amount *R*_*m*_. (*d*) Errors in the estimated time delays, where the terms associated with non-zero delay coefficients *C*_*ij*_ are normalized by the corresponding weights *B*_*ij*_. The coefficients associated with zero terms (without normalization) are shown in the inset. The green and orange curves represent results obtained from networks of size *N*=30 and *N*=60, respectively. All errors are obtained by averaging over 20 independent network realizations. The black arrows indicate the *R*_*m*_ value used to calculate the results in panels (*a*) and (*b*).

We first study the estimation errors with respect to varying data amount, *R*_*m*_. Representative results are shown in figure 3 for *R*_*m*_=0.3 and *R*_*m*_=0.5, where figure 3*a*,*b* are for errors in the weight and time delay estimation, respectively. For small data amount (left column), the gaps between the weights for existent and non-existent links are not well defined, especially for nodes of large degrees. As *R*_*m*_ is increased, the two kinds of weights can be unequivocally distinguished, making possible identification of the existent links (right column). Ensemble-averaged errors in the estimated weights and time delays versus *R*_*m*_ are shown in figure 3*c*,*d*, respectively. Note that, in figure 3*d*, the terms associated with non-zero coefficients *C*_*ij*_ are adjusted by the corresponding weights *B*_*ij*_ to compensate the actual coupling delays and the absolute errors associated with the zero coefficients, as shown in the inset, the averages of the corresponding absolute values of the *c*_*ij*_ terms. A general observation is that the various errors decrease rapidly as the data amount is increased, a prominent feature of compressive sensing.

**Figure 4.**
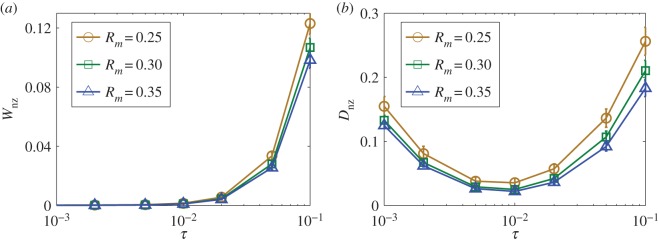
Effect of the amount of time delay on reconstruction performance. For networks with uniform time delays, errors of predicted weights (*a*) and delays (*b*) versus the length of the actual time delay in the network. Shown in (*a*) are the normalized errors associated with the non-zero terms in the weights, for several values of *R*_*m*_. In (*b*), the errors are associated with the time delays of the existent links. Random networks of size *N*=100 and connection probability of *P*=0.04 are used. The results are from three different time-series segments, as marked with different symbols. Each data point is the result of averaging over 10 network realizations.

In our mathematical formulation of the compressive-sensing-based method, the terms containing the time delays are expanded to first order only. The methodology, as it stands now, thus applies to systems with small time delays. To determine an upper bound of the time delay, below which the whole system including various time delays can be reconstructed faithfully, it is necessary to assess the dependence of the estimation errors on the amount of the time delay. Figure 4*a*,*b* show, for the special case of uniform time delay, errors *W*_nz_ and *D*_nz_ versus *τ*, respectively. We see that the weight errors increase monotonically with *τ*, especially for *τ*>10^−2^. However, the time delay errors reach minimum for *τ*≈10^−2^ and begin to increase as *τ* is increased further. For relatively large time delays, the first-order Taylor expansion becomes less accurate, leading to large errors in the weight and time delay estimation. For small delays, the error in *D*_nz_ is due to the finite step size used in integrating the delay differential equations.

**Figure 5.**
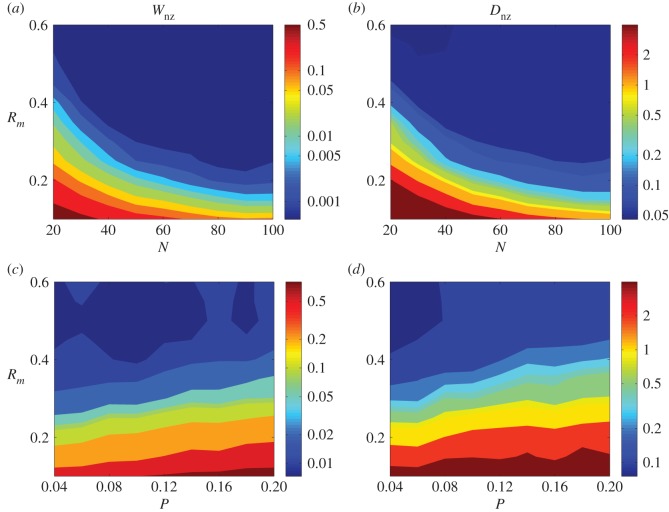
Effect of network size on reconstruction performance. (*a*,*b*) Errors associated with non-zero terms of weights *W*_nz_ and time delays *D*_nz_, respectively, as the network size *N* is increased (left to right), for increasing data amount (bottom to top). The networks are random with fixed link probability *p*=0.04. Cold colours represent small errors. (*c*,*d*) Errors in the weights (*c*) and delays (*d*) versus the connection probability *p* for fixed *N*=30. All results are obtained by averaging over 20 independent network realizations.

How does the performance depend on the network size and other characteristics such as the link density? Figure 5 shows, for random networks of varying size *N* and average connection probability *P*, the errors *W*_nz_ and *D*_nz_. Specifically, for each pair of nodes in the network, their connection probability is given by $p_{ij}∼p_{0}/d_{ij}^{2}$, where *d*_*ij*_ is the distance between them, and *p*_0_ is a normalization constant used to fix the average connection probability as *P*. In this type of ‘normalized’ networks, nodes have a larger tendency to connect to the nearby nodes, as in a real geospatial network. In figure 5*a*,*b*, the network size varies from *N*=30 to *N*=100, while the connection density remains fixed at *P*=0.04. The errors are illustrated using different colours. When the data amount *R*_*m*_ is increased, the errors decrease rapidly and approach a small constant value when *R*_*m*_ exceeds a certain critical value. We find that optimal reconstruction performance can be achieved for smaller values of *R*_*m*_ for networks of larger size than those of smaller size, indicating that accurate reconstruction of a larger network requires relatively smaller ratio of measurements to the number of unknown coefficients, although the absolute data amounts are larger than those for smaller networks. This is so because, as *N* is increased, the density of non-zero terms in the dynamical equations and coupling functions decreases for fixed connection density.

As the connection probability *P* is increased, for example, from 0.02 to 0.2, for a fixed network size (e.g. *N*=30), larger data amount is required for reliable reconstruction, as shown in figure 5*c*,*d*. This is consistent with previous results on reconstruction of complex networks without time delays [35,36], a feature of the compressive-sensing-based method.

### 2.3 Error analysis of triangulation algorithm for node positioning in geophysical space

**Figure 6.**
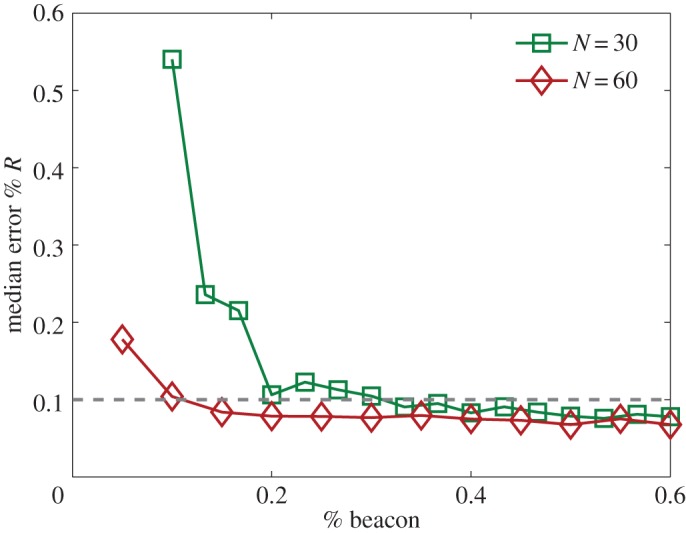
Positioning errors. Normalized positioning error *M*_*r*_, defined as the medium absolute estimated distance error normalized by the distributed length *L*, as a function of the fraction *R*_B_ of the beacon nodes. The networks have the scale-free topology with the average outgoing degree *k*=5. Two values of the network size are used: *N*=30 and *N*=60. The beacon nodes are chosen as those having the largest degrees. The time delays are estimated using the data amount *R*_*m*_=0.5, for which the average error is *D*_nz_≈0.12. The results are obtained by averaging over 10 independent network realizations.

To locate all nodes in a two-dimensional space requires knowledge of the positions of at least three nodes (minimally four nodes in a three-dimensional space). Due to noise, the required number of beacon nodes will generally be larger. Since node positioning is based on time delays estimated from compressive sensing, which contain errors, the number of required beacon nodes is larger than three even in two dimensions. To quantify the positioning accuracy, we use the normalized error *M*_*r*_, defined as the medium distance error between the estimated and actual locations for all nodes (except the beacon nodes), normalized by the distributed length *L*. Figure 6 shows *M*_*r*_ versus the fraction *R*_B_ of the beacon nodes. The reconstruction parameters are chosen such that the errors in the time delay estimation is *D*_nz_≈0.12. For small values of *R*_B_, the positioning errors are large. Reasonable positioning errors are obtained when *R*_B_ exceeds, say, 0.2.

### 2.4 Locating a hidden node in a geospatial network

**Figure 7.**
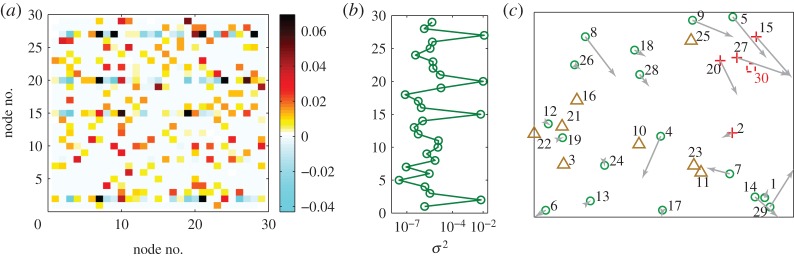
Detection of hidden nodes in geospatial networks. For a random network of *N*=30 nodes, illustration of detecting a hidden node (30). (*a*) Reconstructed time delays using time series from 29 externally accessible nodes. (*b*) Average variance in the reconstructed incoming coupling delays calculated from different segments of the available time series. (*c*) Estimated positions of all accessible nodes in comparison with the respective actual positions, and the location of the hidden node. The triangles denote the beacon nodes, whose positions are known *a priori*. The green circles denote ‘normal’ nodes without any hidden node in their immediate neighbourhoods, while the crosses are direct neighbours of the hidden node. The actual position of the hidden node 30 is marked as a dashed square.

To demonstrate that our compressive-sensing-based approach can be used to ascertain the existence of a hidden node and to estimate its physical location in a geospatial network, we use the model random network in figure 5. A node is regarded as ‘hidden’ when no time series or other type of direct information can be obtained from it. To detect a hidden node, it is necessary to identify its neighbouring nodes [38]. For an externally accessible node, if there is a hidden node in its neighbourhood, the corresponding entry in the reconstructed adjacency matrix will exhibit an abnormally dense pattern or contain meaningless values. In addition, the estimated coefficients for the dynamical and coupling functions of such an abnormal node typically exhibit much larger variations when different data segments are used, in comparison with those associated with normal nodes that do not have hidden nodes in their neighbourhoods. The mathematical formulation of our method to uncover a hidden node can be found in Methods. Initially, there are only 29 time series, one from each of the normal nodes, and it is not known *a priori* that there would be a hidden node in the network. We proceed to reconstruct the network to obtain the estimated weights and time delays, as shown in figure 7*a*. From the results, we find that the connection patterns of some nodes are relatively dense and the values of the weights and time delays are meaningless (e.g. negative values), giving the first clue that these nodes may be the neighbouring nodes of a hidden node. To confirm that this is indeed the case, we divide the available time series into a number of segments under the criterion that the data requirement for reconstruction is satisfied for each segment. As shown in figure 7*b*, we observe extraordinarily large variances in the estimated coefficients associated with the abnormal nodes. Combining results from figure 7*a*,*b*, we can claim with confidence that the four nodes are indeed in the immediate neighbourhood of a hidden node, ascertaining its existence in the network. Our method also works if there are more than one hidden node, given that they do not share common neighbouring nodes.

While the results from figure 7*a*,*b* confirm the existence of a hidden node in the network, its geophysical location is still unknown. Note that each of the neighbouring nodes of the hidden node is connected to a number of ‘normal’ nodes in the network. We can then use the standard triangularization procedure to determine the locations of all the ‘abnormal’ nodes. Since a geospatial random network has the property that nodes tend to connect with physically nearby nodes, we can deduce that the hidden node must be in the geographical vicinity of the abnormal nodes. In the example in figure 7, the hidden node (30, represented by red square) must then stay near its neighbouring nodes (nodes 2, 15, 20 and 27, represented by red crosses), as shown in figure 7*c*, where the normal nodes that are not in the neighbourhood of the hidden node are denoted by green circles.

## 3. Discussion

Given that data are available from a large number of components of a complex networked system which are distributed in a geophysical space, can the network structure be reconstructed, the locations of all nodes be determined and hidden nodes be detected and ascertained? We address these related issues by developing a compressive-sensing-based approach. In particular, assume that time series or signals from nodes in the network are collected at a single location. Our approach enables not only the network topology to be reconstructed, but also the various time delays of the signals from distinct nodes to be estimated. A standard triangularization procedure can then be used to determine the locations of the nodes in the geospatial network, in two-dimensional or three-dimensional space, based on the time delay estimates. We also demonstrate that the existence of a hidden node, from which no signal or time series is externally accessible, can be inferred and ascertained by identifying all nodes in its immediate neighbourhood. The location of the hidden node can then be estimated, as nodes in a geospatial network tend to be locally connected.

We stress that, for data-based reconstruction of complex geospatial networks, a significant challenge is that the available time series are time delayed, due to the finite speed of the physical signals. One unique contribution of this work, which goes beyond those of previous works on compressive-sensing-based reconstruction of complex networks [35–41], is a demonstration that inhomogeneous time delays in a complex network can be estimated reliably using compressive sensing. The information about the time delays allows us to determine the geophysical locations of the nodes. Our approach sheds new light into the general problem of network reconstruction and has potential applications in understanding and exploiting geographically embedded networks.

While we used continuous-time, oscillatory dynamics on networks as a prototypical model, our compressive-sensing-based framework for reconstruction of geospatial networks can be generalized to other types of network dynamical processes. For example, previous works demonstrated that both discrete time maps [35] and evolutionary game dynamics [37] can be formulated as an optimization problem that can be solved by compressive sensing. To include time delays for discrete time maps is straightforward. For evolutionary game dynamics, one can take into account time delays by using a delayed vector for each agent, the entries of which correspond to the time delays between this agent and all other agents in the network. For large networks, the required computation may be demanding. A more serious difficulty may arise when the delays among the agents are substantial, leading to the violation of the sparsity condition required for compressive sensing. To accurately estimate the time delays for complex networks hosting evolutionary game dynamics is thus an open problem at present.

There are still many challenges to apply our method to more general systems. For example, for the linear coupling scheme, we used the Taylor expansion to approximate the delayed coupling functions to the first order in the time delays so that its values can be estimated directly. However, if the coupling function is nonlinear, applying the Taylor expansion will result in complicated terms that involve high orders of the time delays, making extrapolating their values difficult (if not impossible). How to treat nonlinear coupling functions that contain time delays remains to be an open problem. Also, our method can locate nodes only in the Cartesian space, as the distances are inferred from the time delays information through a triangulation algorithm that is specifically designed to deal with the Cartesian space. Extending the methodology to curved space is an interesting issue but it is open at present. Another difficulty is that high-dimensional nodal dynamical equations may not be sparsely expressed through a Taylor expansion. In this case, we can either choose alternative expansion techniques, such as Fourier series, or test alternative observation variables to obtain suitable response functions. For example, in the particular case of reconstructing stochastic evolutionary game networks [37,38], the dynamical processes are not described by nonlinear differential equations but are defined in terms of a set of stochastic game rules. At every time step, each agent chooses its strategy in a random manner. A workable set of observation variables can be the pay-offs of all the agents. It was demonstrated [37,38] that the network structure under stochastic game rules can still be constructed with high accuracy, based on compressive sensing.

## 4. Methods

### 4.1 Mathematical framework for reconstructing coupled oscillator networks with time delay

As proof of principle, we present our reconstruction framework using continuous time oscillator networks. There are *N* oscillators in the network, and the dynamical process on each node is described by a set of coupled ordinary differential equations. (A similar framework can be formulated for other types of dynamical processes, such as evolutionary games [37].) The *m*-dimensional state variable of node *i* is written as equation (2.2). To derive equation (2.3), we assume linear coupling functions and causality so that all *τ*_*ij*_ (*i*,*j*=1,…,*N*) are positive (for simplicity). We regroup all terms directly associated with node *i* into ${\text{F}}_{i}^{\prime }[{\text{x}}_{i}(t)]$, where

4.1 $${\text{F}}_{i}^{\prime }[{\text{x}}_{i}(t)]\equiv {\text{F}}_{i}[{\text{x}}_{i}(t)]-{\text{x}}_{i}(t)\cdot \sum_{j=1,j\neq i}^{N}{\text{W}}_{ij},$$

and we expand ${\text{F}}_{i}^{\prime }[{\text{x}}_{i}(t)]$ into the following series form:

4.2 $${\text{F}}_{i}^{\prime }[{\text{x}}_{i}(t)]=\sum_{\gamma }{\tilde{\alpha }}^{\left(\gamma \right)}\cdot {\tilde{\text{g}}}^{\left(\gamma \right)}[{\text{x}}_{i}(t)],$$

where ${\tilde{\text{g}}}^{\left(\gamma \right)}[{\text{x}}_{i}(t)]$ represents a suitably chosen set of orthogonal and complete base functions such that the coefficients ${\tilde{\alpha }}^{\left(\gamma \right)}$ are sparse. To proceed, we approximate **x**_*j*_(*t*−*τ*_*ij*_) as

4.3 $${\text{x}}_{j}(t-\tau_{ij})\approx {\text{x}}_{j}(t)-\tau_{ij}{\dot{\text{x}}}_{j}(t),$$

so all the coupling terms with inhomogeneous time delays associated with node *i* can be written as

4.4 $${\left[\sum_{j=1,j\neq i}^{N}{\text{W}}_{ij}{\text{x}}_{j}(t-\tau_{ij})\right]}_{p}\equiv \sum_{j=1,j\neq i}^{N}\left[{\text{b}}_{ij}{\text{x}}_{j}(t)+{\text{c}}_{ij}{\dot{\text{x}}}_{j}(t)\right],$$

where **b**_*ij*_=**W**_*ij*_ and **c**_*ij*_=−**W**_*ij*_*τ*_*ij*_. Equation (2.2) can then be written in the compact form as equation (2.3), which is a set of linear equations, where ${\tilde{\alpha }}^{\left(\gamma \right)}$, **b**_*ij*_ and **c**_*ij*_ are to be determined. If the unknown coefficient vectors can be reconstructed accurately, we will have complete information about the nodal dynamics as represented by **F**^′^[**x**(*t*)], the topology and interacting weights of the underlying network as represented by **W**_*ij*_, as well as the time delays associated with the non-zero links because of the relations **W**_*ij*_=**b**_*ij*_ and *τ*_*ij*_=−**c**_*ij*_/**b**_*ij*_. However, if the coupling form is nonlinear, the relationship between the delay term **c**_*ij*_ and the time delays *τ*_*ij*_ would be hard to interpret, especially when the exact coupling form is not known.

As an illustrative example, we consider the case where each individual nodal dynamical system is three-dimensional with variables *x*, *y* and *z*. For the first component *x*_*i*_ of node *i*, we have the following series expansion at time *t*:

4.5 $$\begin{array}{rl} \dot{x_{i}} & ={\left({\tilde{a}}_{i}\right)}_{000}\cdot x_{i}^{0}y_{i}^{0}z_{i}^{0}+\cdots +{\left({\tilde{a}}_{i}\right)}_{333}\cdot x_{i}^{3}y_{i}^{3}z_{i}^{3}+{\left(b_{i1}\right)}_{1}x_{1}+{\left(b_{i1}\right)}_{2}y_{1}+{\left(b_{i1}\right)}_{3}z_{1}+\cdots +{\left(b_{iN}\right)}_{1}x_{N}\\ & \;+{\left(b_{iN}\right)}_{2}y_{N}+{\left(b_{iN}\right)}_{3}z_{N}+{\left(c_{i1}\right)}_{1}{\dot{x}}_{1}+{\left(c_{i1}\right)}_{2}{\dot{y}}_{1}+{\left(c_{i1}\right)}_{3}{\dot{z}}_{1}+\cdots +{\left(c_{iN}\right)}_{1}{\dot{x}}_{N}+{\left(c_{iN}\right)}_{2}{\dot{y}}_{N}+{\left(c_{iN}\right)}_{3}{\dot{z}}_{N}, \end{array}$$

where *b*_*ii*_ and *c*_*ii*_ are excluded. The formula contains three parts: the power series of isolated nodal dynamics with coefficients ${\tilde{a}}_{i}$, terms of all other coupled nodes’ variables with coefficients *b*_*ij*_, and terms of derivatives of the coupled nodes as represented by *c*_*ij*_. Assuming that measurements *x*_*i*_(*t*), *y*_*i*_(*t*) and *z*_*i*_(*t*) at a set of time instants *t*_1_,*t*_2_,…,*t*_*w*_ are available, we write

4.6 $$\begin{array}{rl} {\text{A}}_{i}(t) & =[x_{i}{\left(t\right)}^{0}y_{i}{\left(t\right)}^{0}z_{i}{\left(t\right)}^{0},\ldots ,x_{i}{\left(t\right)}^{3}y_{i}{\left(t\right)}^{3}z_{i}{\left(t\right)}^{3}], \end{array}$$

4.7 $$\begin{array}{rl} {\text{B}}_{i}(t) & =[x_{1}(t),\ldots ,x_{N}(t),y_{1}(t),\ldots ,y_{N}(t),z_{1}(t)\ldots ,z_{N}(t)] \end{array}$$

4.8 $$\begin{array}{rl} \;\text{and}\;\;{\text{C}}_{i}(t) & =[{\dot{x}}_{1}(t),\ldots ,{\dot{x}}_{N}(t),{\dot{y}}_{1}(t),\ldots ,{\dot{y}}_{N}(t),{\dot{z}}_{1}(t)\ldots ,{\dot{z}}_{N}(t)] \end{array}$$

to obtain a compact expression

4.9 $$\text{X}=\text{G}\cdot {\text{a}}_{i}+\xi ,$$

where

4.10 $$\text{X}=\left(\begin{array}{c} {\dot{x}}_{i}(t_{1})\\ {\dot{x}}_{i}(t_{2})\\ \vdots \\ {\dot{x}}_{i}(t_{w}) \end{array}\right)=\left(\begin{array}{ccc} {\text{A}}_{i}(t_{1}) & {\text{B}}_{i}(t_{1}) & {\text{C}}_{i}(t_{1})\\ {\text{A}}_{i}(t_{2}) & {\text{B}}_{i}(t_{2}) & {\text{C}}_{i}(t_{2})\\ \vdots & \vdots & \vdots \\ {\text{A}}_{i}(t_{w}) & {\text{B}}_{i}(t_{w}) & {\text{C}}_{i}(t_{w}) \end{array}\right)\left(\begin{array}{c} {\tilde{\text{a}}}_{i}\\ {\text{b}}_{i}\\ {\text{c}}_{i} \end{array}\right)+\xi ,$$

and *ξ* represents the error introduced by the series approximation.

In general, the connection pattern of a complex network is far sparser than the all-to-all coupling configuration, so typically most elements of ${\left[{\tilde{\text{a}}}_{i},{\text{b}}_{i},{\text{c}}_{i}\right]}^{T}$ are zero. In addition, the error *ξ* is small and can be regarded as a noise term.

### 4.2 Compressive sensing algorithm in the presence of noise

The compressive sensing algorithm can be used to solve a sparse vector **a** from the ill-conditioned linear equation under noise: **X**=**G**⋅**a**+*ξ*, where *ξ* is a random process. Reliable recovery of the sparse vector **a** can be achieved [29,30,33] by solving the following *l*_1_ regularization problem:

4.11 $$\text{min}\;∥\text{a}{∥}_{l_{1}},\;\text{subject to}\;∥\text{G}\cdot \text{a}-\text{X}\;{∥}_{l_{2}}\leq \varepsilon ,$$

where the *l*_1_ norm for a vector **x** is defined as $∥\text{x}{∥}_{l_{1}}=\sum_{i=1}^{n}|x_{i}|$ (its *l*_2_ norm is $∥\text{x}{∥}_{l_{2}}=\sum_{i=1}^{n}|x_{i}^{2}|$) and *ε* is a threshold value determined by the noise amplitude. The reconstructed vector $\bar{\text{a}}$ lies within the range: $∥\bar{\text{a}}-\text{a}∥\leq C\cdot \varepsilon$, where *C* is a constant.

In order to apply the compressive sensing algorithm to solve equation (4.10), it is necessary to normalize each column of the matrix **G** by the *L*_2_ norm of that column: (**G**^′^)_*ij*_=(**G**)_*ij*_/*L*_2_(*j*) with $L_{2}(j)=\sqrt{\sum_{i=1}^{M}{\left[{\left(\text{G}\right)}_{ij}\right]}^{2}}$. We thus have **X**=**G**^′^⋅**u**^′^ with **u**^′^=**u***L*_2_. After **u**^′^ is determined through some standard compressive sensing algorithm, the coefficients **u** are given by **u**^′^/*L*_2_. Substituting **a**_*i*_, **b**_*ij*_ and **c**_*ij*_ back into equation (2.3), we obtain the nodal dynamics, coupling weights and the delays associated with the first dynamical variable of node *i*. For the remaining two variables for this node and all variables for other nodes in the network, a similar procedure can be followed.

### 4.3 Triangle localization method

Given the positions of *k* reference nodes (or beacon nodes) (*x*_*k*_,*y*_*k*_), and their distances *d*_*i*,1_,*d*_*i*,2_,…*d*_*i*,*k*_ to the target node *i*, we can calculate the position of node *i* using the triangular localization method [44], for *k* larger than the space dimension. In general, we will need to solve the least-squares optimization problem **H**⋅**x**_*i*_=**b**, where **x**_*i*_=[*x*_*i*_,*y*_*i*_]^T^ is the position of node *i*, and **H**=[**x**_1_,**x**_2_,…,**x**_*k*_]^T^ is the position vector corresponding to the set of beacon nodes, where **b**=0.5×[*D*_1_,*D*_2_,…,*D*_*k*_]^T^ and $D_{k}=d_{ik}^{2}-y_{k}^{2}+x_{k}^{2}$.

To locate the positions of all nodes in the network, we start with a small set of beacon nodes whose actual positions are known and the distances associated with all links in the network. Initially, we can locate the nodes that are connected to at least three nodes in the set of beacon nodes, insofar as the three reference nodes are not located on a straight line. When this is done, the newly located nodes can be added into the set of reference nodes and the neighbouring nodes can be located through the new set of beacon nodes. We iterate this process until the positions of all nodes are determined or no more qualified neighbouring nodes can be found. For a general network, such initial beacon sets may not be easily found. A special case is scale-free networks, for which the initial beacon set can be chosen as the nodes with the largest degrees. For a random network, we can also choose the nodes of the largest degree as the initial beacon node set and use a larger beacon set to locate most of the nodes in the network. For an arbitrary topology, we offer the following simple method to select the set of beacon nodes: we estimate the distances from one node to all other unconnected nodes using the weighted shortest distance and then proceed with the triangular localization algorithm. There are alternative localization algorithms based on given distances, for example, the multidimensional scaling method [52,53].

### 4.4 Asynchronous data collection

In real applications, the requirement to collect time series simultaneously will usually not be met. Consider the typical situation where the data are collected from a fixed external node, denoted by *s*, which has varying distances to the signal sources. The signals that arrive at the external node at time *t* were actually sent out by the sources at time *t*−*τ*_*is*_, where *τ*_*is*_ is the varying transmission delay associated with the distances from node *i* to *s*. In general, *τ*_*is*_ is unknown *a priori* because the location of node *i* needs to be determined.

In the reconstruction of the dynamical process and connections associated with node *i*, the time series substituted into equation (2.3) are in fact *x*_*i*_(*t*−*τ*_*is*_) and *x*_*j*_(*t*−*τ*_*js*_), for *j*=1,2,…,*N* and *j*≠*i*. The delay coupling terms can approximated as

4.12 $${\text{x}}_{j}(t-\tau_{is}-\tau_{ij})={\text{x}}_{j}(t-\tau_{js}-\tau_{is}-\tau_{ij}+\tau_{js})\approx {\text{x}}_{j}(t-\tau_{js})-\tau_{ij}^{\prime }{\dot{\text{x}}}_{j}(t-\tau_{js}),$$

where *τ*_*ij*_ is the actual delay and $\tau_{ij}^{\prime }$ is the estimated delay. From Taylor expansion, we have $\tau_{ij}^{\prime }=\tau_{is}-\tau_{js}+\tau_{ij}$. Similarly, for node *j*, the estimated delay is $\tau_{ji}^{\prime }=\tau_{js}-\tau_{is}+\tau_{ji}$. Because *τ*_*ij*_=*τ*_*ji*_, we can eliminate the effect of *τ*_*is*_ and *τ*_*js*_ by averaging the two estimated delays, as $\tau_{ij}=\tau_{ji}=(\tau_{ij}^{\prime }+\tau_{ji}^{\prime })/2$. After we obtain the time-delay matrix $\left\{\tau_{ij}^{\prime }\right\}$, we can convert its elements into the actual elements so as to obtain the corresponding distances {*d*_*ij*_}.

### 4.5 Locating a hidden node in a random geospatial network

We first reconstruct the network using our compressive sensing framework, treating the system as if there was no hidden node. As demonstrated in figure 7, the neighbouring nodes of the hidden node tend to exhibit abnormally dense connection patterns. We then use multiple time-series segments to calculate the variance in the reconstructed coefficient vectors for all nodes. In particular, the variance *σ*_*i*_ associated with node *i* is defined as

4.13 $$\sigma_{i}=\sqrt{\frac{1}{T}\sum_{t=1}^{T}\frac{1}{N}\sum_{k=1}^{N}{\left(w_{ik}-{\tilde{w}}_{ik}\right)}^{2}},$$

where *T* is the number of data segments used, *N* the network size and ${\tilde{w}}_{ij}$ the average weights over *T* realizations. The variance associated with the time delays can be calculated in a similar way. The neighbouring nodes of the hidden node are those with abnormally dense connection patterns *and* significantly larger variances than others.

If there are more than one hidden node but they are not directly linked, i.e. they do not share any neighbouring nodes, we can use the cancellation method developed in our previous work [39] to ascertain the number of hidden nodes. The first step is to identify all neighbouring nodes. The second step is to check if the neighbouring nodes are connected to the same hidden node by calculating the cancellation factor for any pair of neighbouring nodes. If they are connected to the same hidden node, the cancellation factor is the ratio of their coupling strength to the common hidden node, which is typically non-zero; otherwise they are connected to different hidden nodes. The third step is to repeat this process for all possible pairs of the identified neighbouring nodes. This procedure allows us to classify the neighbouring nodes into different groups, each containing a hidden node.

## References

[1] Gruen S, Diesmann M, Aertsen A 2002 Unitary events in multiple single neuron spiking activity. I. Detection and significance. Neural Comp. 14, 43–80. (doi:10.1162/089976602753284455) 10.1162/089976602753284455 11747534

[2] Gütig R, Aertsen A, Rotter S 2002 Statistical significance of coincident spikes: count-based versus rate-based statistics. Neural Comp. 14, 121–153. (doi:10.1162/089976602753284473) 10.1162/089976602753284473 11747536

[3] Gardner TS, di Bernardo D, Lorenz D, Collins JJ 2003 Inferring genetic networks and identifying compound mode of action via expression profiling. Science 301, 102–105. (doi:10.1126/science.1081900) 12843395 10.1126/science.1081900

[4] Pipa G, Grün S 2003 Non-parametric significance estimation of joint-spike events by shuffling and resampling. Neurocomputing 52, 31–37. (doi:10.1016/S0925-2312(02)00823-8)

[5] Brovelli A, Ding M, Ledberg A, Chen Y, Nakamura R, Bressler SL 2004 Beta oscillations in a large-scale sensorimotor cortical network: directional influences revealed by Granger causality. Proc. Natl Acad. Sci. USA 101, 9849–9854. (doi:10.1073/pnas.0308538101) 15210971 10.1073/pnas.0308538101 PMC470781

[6] Bongard J, Lipson H 2007 Automated reverse engineering of nonlinear dynamical systems. Proc. Natl Acad. Sci. USA 104, 9943–9948. (doi:10.1073/pnas.0609476104) 17553966 10.1073/pnas.0609476104 PMC1891254

[7] Timme M 2007 Revealing network connectivity from response dynamics. Phys. Rev. Lett. 98, 224101 (doi:10.1103/PhysRevLett.98.224101) 17677845 10.1103/PhysRevLett.98.224101

[8] Tang WKS, Yu M, Kocarev L 2007 Identification and monitoring of biological neural network. In *IEEE Int. Symp. on Circuits and Systems (ISCAS)*, pp. 2646–2649. Piscataway, NJ: IEEE (doi:10.1109/ISCAS.2007.377957)

[9] Napoletani D, Sauer TD 2008 Reconstructing the topology of sparsely connected dynamical networks. Phys. Rev. E 77, 026103 (doi:10.1103/PhysRevE.77.026103) 10.1103/PhysRevE.77.026103 18352086

[10] Sontag E 2008 Network reconstruction based on steady-state data. Essays Biochem. 45, 161–176. (doi:10.1042/bse0450161) 18793131 10.1042/BSE0450161

[11] Wang WX, Chen QF, Huang L, Lai YC, Harrison M 2009 Scaling of noisy fluctuations in complex networks and applications to network prediction. Phys. Rev. E 80, 016116 (doi:10.1103/PhysRevE.80.016116) 10.1103/PhysRevE.80.016116 19658783

[12] Ren J, Wang WX, Li B, Lai YC 2010 Noise bridges dynamical correlation and topology in coupled oscillator networks. Phys. Rev. Lett. 104, 058701 (doi:10.1103/PhysRevLett.104.058701) 20366800 10.1103/PhysRevLett.104.058701

[13] Yuan Y, Stan GB, Warnick S, Goncalves J 2010 Robust dynamical network reconstruction. In *49th IEEE Conf. on Decision and Control (CDC)*, pp. 810–815. Piscataway, NJ: IEEE (doi:10.1109/CDC.2010.5717657)

[14] Levnajić Z, Pikovsky A 2011 Network reconstruction from random phase resetting. Phys. Rev. Lett. 107, 034101 (doi:10.1103/PhysRevLett.107.034101) 21838361 10.1103/PhysRevLett.107.034101

[15] Hempel S, Koseska A, Kurths J, Nikoloski Z 2011 Inner composition alignment for inferring directed networks from short time series. Phys. Rev. Lett. 107, 054101 (doi:10.1103/PhysRevLett.107.054101) 21867072 10.1103/PhysRevLett.107.054101

[16] Shandilya SG, Timme M 2011 Inferring network topology from complex dynamics. New J. Phys. 13, 013004 (doi:10.1088/1367-2630/13/1/013004)

[17] Yu D, Parlitz U 2011 Inferring network connectivity by delayed feedback control. PLoS ONE 6, e24333 (doi:10.1371/journal.pone.0024333) 21969856 10.1371/journal.pone.0024333 PMC3182170

[18] Pan W, Yuan Y, Stan GB 2012 Reconstruction of arbitrary biochemical reaction networks: a compressive sensing approach. In *51st IEEE Annual Conf. on Decision and Control (CDC)*, pp. 2334–2339. Piscataway, NJ: IEEE (doi:10.1109/CDC.2012.6426216)

[19] Wang WX, Ren J, Lai YC, Li B 2012 Reverse engineering of complex dynamical networks in the presence of time-delayed interactions based on noisy time series. Chaos 22, 033131 (doi:10.1063/1.4747708) 23020470 10.1063/1.4747708

[20] Berry T, Hamilton F, Peixoto N, Sauer T 2012 Detecting connectivity changes in neuronal networks. J. Neurosci. Methods 209, 388–397. (doi:10.1016/j.jneumeth.2012.06.021) 22771714 10.1016/j.jneumeth.2012.06.021

[21] Stetter O, Battaglia D, Soriano J, Geisel T 2012 Model-free reconstruction of excitatory neuronal connectivity from calcium imaging signals. PLoS Comput. Biol. 8, e1002653 (doi:10.1371/journal.pcbi.1002653) 22927808 10.1371/journal.pcbi.1002653 PMC3426566

[22] Hamilton F, Berry T, Peixoto N, Sauer T 2013 Real-time tracking of neuronal network structure using data assimilation. Phys. Rev. E 88, 052715 (doi:10.1103/PhysRevE.88.052715) 10.1103/PhysRevE.88.052715 24329304

[23] Zhou D, Xiao Y, Zhang Y, Xu Z, Cai D 2013 Causal and structural connectivity of pulse-coupled nonlinear networks. Phys. Rev. Lett. 111, 054102 (doi:10.1103/PhysRevLett.111.054102) 23952403 10.1103/PhysRevLett.111.054102

[24] Timme M, Casadiego J 2014 Revealing networks from dynamics: an introduction. J. Phys. A. Math. Theor. 47, 343001 (doi:10.1088/1751-8113/47/34/343001)

[25] Donges J, Zou Y, Marwan N, Kurths J 2009 The backbone of the climate network. Europhys. Lett. 87, 48007 (doi:10.1209/0295-5075/87/48007)

[26] Chan J, Holmes A, Rabadan R 2010 Network analysis of global influenza spread. PLoS Comput. Biol. 6, e1001005 (doi:10.1371/journal.pcbi.1001005) 21124942 10.1371/journal.pcbi.1001005 PMC2987833

[27] Makarov VA, Panetsos F, Feo O 2005 A method for determining neural connectivity and inferring the underlying network dynamics using extracellular spike recordings. J. Neurosci. Methods 144, 265–279. (doi:10.1016/j.jneumeth.2004.11.013) 15910987 10.1016/j.jneumeth.2004.11.013

[28] Yao C, Bollt EM 2007 Modeling and nonlinear parameter estimation with Kronecker product representation for coupled oscillators and spatiotemporal systems. Phys. D 227, 78–99. (doi:10.1016/j.physd.2006.12.006)

[29] Donoho D 2006 Compressed sensing. IEEE Trans. Info. Theory 52, 1289–1306. (doi:10.1109/TIT.2006.871582)

[30] Candès E, Romberg J, Tao T 2006 Stable signal recovery from incomplete and inaccurate measurements. Commun. Pure Appl. Math. 59, 1207–1223. (doi:10.1002/cpa.20124)

[31] Candes` E 2006 Compressive sampling. In *Proc. Int. Congress of Mathematicians*, vol. 3, pp. 1433–1452. Zürich, Switzerland: European Mathematical Society.

[32] Candes` E, Wakin M 2008 An introduction to compressive sampling. IEEE Signal Process. Mag. 25, 21–30. (doi:10.1109/MSP.2007.914731)

[33] Candès E, Romberg J, Tao T 2006 Robust uncertainty principles: exact signal reconstruction from highly incomplete frequency information. IEEE Trans. Info. Theory 52, 489–509. (doi:10.1109/TIT.2005.862083)

[34] Baraniuk RG 2007 Compressed sensing. IEEE Signal Process. Mag. 24, 118–121. (doi:10.1109/MSP.2007.4286571)

[35] Wang WX, Yang R, Lai YC, Kovanis V, Grebogi C 2011 Predicting catastrophes in nonlinear dynamical systems by compressive sensing. Phys. Rev. Lett. 106, 154101 (doi:10.1103/PhysRevLett.106.154101) 21568562 10.1103/PhysRevLett.106.154101 PMC3657682

[36] Wang WX, Yang R, Lai YC, Kovanis V, Harrison MAF 2011 Time-series-based prediction of complex oscillator networks via compressive sensing. Europhys. Lett. 94, 48006 (doi:10.1209/0295-5075/94/48006)

[37] Wang WX, Lai YC, Grebogi C, Ye JP 2011 Network reconstruction based on evolutionary-game data via compressive sensing. Phys. Rev. X 1, 021021 (doi:10.1103/PhysRevX.1.021021)

[38] Su RQ, Wang WX, Lai YC 2012 Detecting hidden nodes in complex networks from time series. Phys. Rev. E 85, 065201 (doi:10.1103/PhysRevE.85.065201) 10.1103/PhysRevE.85.065201 23005153

[39] Su RQ, Lai YC, Wang X, Do YH 2014 Uncovering hidden nodes in complex networks in the presence of noise. Sci. Rep. 4, 3944 (doi:10.1038/srep03944) 24487720 10.1038/srep03944 PMC3909906

[40] Su RQ, Ni X, Wang WX, Lai YC 2012 Forecasting synchronizability of complex networks from data. Phys. Rev. E 85, 056220 (doi:10.1103/PhysRevE.85.056220) 10.1103/PhysRevE.85.056220 23004856

[41] Shen ZS, Wang WX, Fan Y, Di ZR, Lai YC 2014 Reconstructing propagation networks with natural diversity and identifying hidden source. Nat. Commun. 5, 4323 (doi:10.1038/srep11404) 25014310 10.1038/ncomms5323 PMC4104449

[42] Wu X 2008 Synchronization-based topology identification of weighted general complex dynamical networks with time-varying coupling delay. Phys. A 387, 997–1008. (doi:10.1016/j.physa.2007.10.030)

[43] Husmeier D 2003 Sensitivity and specificity of inferring genetic regulatory interactions from microarray experiments with dynamic Bayesian networks. Bioinformatics 19, 2271–2282. (doi:10.1093/bioinformatics/btg313) 14630656 10.1093/bioinformatics/btg313

[44] Sayed A, Tarighat A, Khajehnouri N 2005 Network-based wireless location: challenges faced in developing techniques for accurate wireless location information. IEEE Signal Process. Mag. 22, 24–40. (doi:10.1109/MSP.2005.1458275)

[45] Moon IC, Carley K 2007 Modeling and simulating terrorist networks in social and geospatial dimensions. IEEE Intell. Syst. 22, 40–49. (doi:10.1109/MIS.2007.4338493)

[46] Li C, Chen G 2004 Synchronization in general complex dynamical networks with coupling delays. Phys. A 343, 263–278. (doi:10.1016/j.physa.2004.05.058)

[47] Dhamala M, Jirsa V, Ding M 2004 Enhancement of neural synchrony by time delay. Phys. Rev. Lett. 92, 074104 (doi:10.1103/PhysRevLett.92.074104) 14995856 10.1103/PhysRevLett.92.074104

[48] Gross T, Rudolf L, Levin S, Dieckmann U 2009 Generalized models reveal stabilizing factors in food webs. Science 325, 747–750. (doi:10.1126/science.1173536) 19661430 10.1126/science.1173536

[49] Barabási AL, Albert R 1999 Emergence of scaling in random networks. Science 286, 509–512. (doi:10.1126/science.286.5439.509) 10521342 10.1126/science.286.5439.509

[50] Erdös P, Rényi A 1959 On random graphs I. Publ. Math. Debrecen 6, 290–291.

[51] Bellen A, Zennaro M 2013 Numerical methods for delay differential equations. Oxford, UK: Oxford University Press.

[52] Shang Y, Ruml W 2004 Improved MDS-based localization. In *23rd Annual Joint Conf. of the IEEE Computer and Communications Societies*, vol. 4, pp. 2640–2651. Piscataway, NJ: IEEE (doi:10.1109/INFCOM.2004.1354683)

[53] Shang Y, Ruml W, Zhang Y, Fromherz MP 2003 Localization from mere connectivity. In *Proc. 4th ACM Int. Symp. on Mobile ad hoc Networking & Computing*, pp. 201–212. New York, NY: ACM (doi:10.1145/778415.778439)

